# Interdisciplinary clinicians’ attitudes, challenges, and success strategies in providing care to transgender people: a qualitative descriptive study

**DOI:** 10.1186/s12913-022-08517-x

**Published:** 2022-09-08

**Authors:** Kodiak Ray Sung Soled, Oscar E. Dimant, Jona Tanguay, Ronica Mukerjee, Tonia Poteat

**Affiliations:** 1 Department of Population Medicine, Harvard Medical School, Harvard Pilgrim Health Care Institute, Boston, MA USA; 2 Columbia University School of Nursing, New York, NY USA; 3 Rutgers-New Jersey, Medical School, Kessler Institute for Rehabilitation, Newark, NJ USA; 4 Whitman-Walker Health, Washington, District of Columbia USA; 5 Department of Social Medicine, University of North Carolina, Chapel Hill, NC USA

**Keywords:** Transgender persons, Health services accessibility, Healthcare disparities, Primary health care, Qualitative Studies, Health personnel

## Abstract

**Background:**

Access to clinicians competent in transgender health remains a significant barrier and contributor toward health inequity for transgender people. Studies on access and barriers to care have predominantly evaluated transgender patients’ perceptions, but scant research has included the perspectives of clinicians.

**Aims:**

We conducted a qualitative study to explore how clinicians (meaning physicians and advanced practice providers, in this paper) in the United States: (1) attain and utilize information, (2) perceive barriers and facilitators, and (3) understood gaps in their professional training, in regard to practicing transgender health care.

**Methods:**

A Qualitative Descriptive approach guided our conventional content analysis of field notes and interviews with clinicians within a parent study that explored health care access among transgender adults. Transcripts were coded into meaning units that were iteratively abstracted into themes. Standard measures were performed to promote the trustworthiness of the analysis and reduce bias.

**Results:**

Participants (*n* = 13) consisted of physicians (*n* = 8), physician assistants (*n* = 3), and nurse practitioners (*n* = 2). The majority were women (*n* = 11), identified as White (*n* = 9)*,* cisgender *(n* = 13*),* and ages ranged from 31 – 58 years. Five main themes were identified: (1) Knowledge Acquisition: Formal and Informal Pathways to Competency; (2) Perceived Challenges and Barriers: *I didn’t know what I was doing*; (3) Power to Deny: Prescriptive Authority and Gatekeeping; (4) Stigma: *This is really strange, and I can’t really understand it*; (5) Reflections: Strategies for Success, Rewards, and Personal Motivations.

**Discussion:**

Clinicians gained a sense of comfort and competence with mentorship, self-directed learning, clinical experience, and person-centered, harm-reduction approaches. Stigma, bias, and structural-level factors were barriers to providing care. This study offers a unique perspective of clinicians’ motivations and strategies for providing gender-affirming care and elucidates how stigma impacts the delivery of gender-affirming care.

**Supplementary Information:**

The online version contains supplementary material available at 10.1186/s12913-022-08517-x.

## Introduction

Transgender people include individuals whose gender identity differs from their assigned sex at birth. Approximately one-third of the 1.6 million transgender people in the United States (US) [1] suffer negative encounters in health care settings due to clinician (physicians and advanced practice providers) knowledge deficits, verbal harassment, and refusal to treat [2], and transgender people have described a range of affirming and stigmatizing clinician behaviors in the US healthcare system [3, 4]. A scarcity of clinicians competent in transgender health is a significant barrier to accessing health care [5], contributing to delays in receiving care and unmet health needs [6]. Up to one-third of transgender people in the US avoid or delay care out of fear of discrimination, with four times greater odds of delaying care when they previously had to teach a clinician about transgender health care [7]. Even worse, fear of discrimination and subsequent postponement of care may be associated with worse physical and mental health for this population [8] .

Research conducted in the US and other Westernized countries (e.g., Canada) suggests that insufficient training, knowledge gaps, challenges accessing information, and stigmatizing beliefs perpetuate clinicians’ inability to provide gender-affirming care and consequently adverse health outcomes [9–13]. Stigma – “a process of othering, blaming, and shaming [14]” – at structural and institutional levels additionally contributes to insufficient training and clinical guidelines for clinicians to provide evidenced-based gender-affirming care [13]. Despite increased educational initiatives including curriculum guidelines from major US health professional associations [15–17], barriers to care and health disparities persist. Moreover, transphobia may mitigate the impact of increased hours of education [18], the quantity and quality of curricula may be insufficient [19–21], curricular efforts without experiential learning may be inadequate to overcome anti-transgender attitudes [22], and emphasis on sexual minority (e.g., lesbian, gay, and bisexual) health care may overshadow transgender-specific topics [23, 24].

Compared to research on patient and student perceptions or assessments of competence, knowledge, and attitudes, scant research has investigated clinicians’ perceptions regarding the barriers and facilitators of providing gender-affirming care [5]. Yet, clinicians’ perspectives are vital to identifying solutions and interventions that will be acceptable and specific in addressing clinician-level factors in providing gender-affirming care. Among the few US and Canadian-based studies that have addressed this, clinician challenges have included: [25]not knowing where or from whom to obtain knowledge hindering competency [25, 26]; limited patient experience hindering confidence [26]; and appointment time constraints hindering management of concurrent health conditions [27]. Yet, there are still many unanswered questions in the literature such as: what additional factors impact clinicians’ ability to provide gender-affirming care, how is clinical competence developed in this area, and what is needed (e.g., training, certification, mentorship) to promote clinicians’ willingness and ability to provide gender-affirming care.

Thus, to gain a deeper understanding of clinicians’ perspectives on providing gender-affirming care, we conducted a secondary analysis of an existing qualitative study. We triangulated gaps in the literature with questions posed in the parent study’s interview guide to craft our research question: to explore how physicians, nurse practitioners, and physician assistants in an East Coast city of the US: (1) attained and utilized information, (2) perceived barriers and facilitators, and (3) understood gaps in their professional training, in regard to practicing transgender health care.

## Methods

We conducted a Qualitative Descriptive [28] study using a naturalistic perspective and a conventional content analysis [29] of field notes and in-depth interviews with 13 clinicians. Qualitative, in-depth interviews are appropriate when seeking to understand an experience from a personal perspective [30], and a qualitative descriptive approach is useful for gaining insights regarding a poorly understood phenomenon to develop an intervention – particularly in healthcare.[31] These interviews took place within the context of a larger study of clinicians (*n* = 12) and transgender people (*n* = 55) that explored institutional factors associated with health care access and HIV risks among transgender adults in a small, mid-Atlantic city [13]. This analysis seeks to answer critical questions about clinician knowledge and perspectives not addressed in the parent study [32]. The format of this paper follows the 32-item consolidated criteria for reporting qualitative studies (COREQ, see additional file 1) [33].

### Recruitment and data collection in the parent study

A purposive sample of clinicians was recruited via email from health care settings known to provide care for transgender people. Clinicians who were 18 years of age or older, worked in the metropolitan area, and provided care to at least one transgender person within the prior year were eligible to participate.

Interviews were conducted using a semi-structured interview guide (see additional file 2) from January to July 2011 and took place in settings based on the participants’ preference: the project office, the clinician’s office or home, a car, a cafe, or the telephone. Interviews lasted between 30 and 90 min with a mean duration of 62 min. Verbal consent was obtained from all participants. Interviews were audiotaped and transcribed verbatim. Field notes were handwritten immediately following each interview, then typed and added to transcripts. To enhance confidentiality, no written consent forms were used. Individuals were read the consent form and provided time to ask questions before providing their verbal consent. The Institutional Review Board at Johns Hopkins School of Public Health approved the parent study (IRB00007908), including oral consent, and an administrative amendment was obtained for this secondary analysis.

### Research team and reflexivity

The senior author conducted the majority of interviews (*n* = 11), and a trained graduate research assistant conducted the remaining two interviews. The senior author had a collegial relationship with half of the clinicians (*n* = 6), but they chose to be interviewed by her rather than the research assistant. Both the senior author and her research assistant are cisgender women of color (Black and Asian American, respectively) who have extensive experience working with transgender communities. The study was grounded in the community through establishing two community advisory boards that comprised of transgender men and women per community request. No members identified as nonbinary, but gender diverse identities were less common at the time of data collection than they are today. Advisory members provided feedback into the development of study materials, assisted with recruitment, and supported the interpretation of preliminary findings for the parent study. Results from that study have been published, and details are available elsewhere [13].

The first author is a White, cisgender queer woman and LGBTQ + health researcher with a background in nursing. She engaged in a reflexive practice throughout the analytic process that included bracketing for preconceptions and biases stemming from her sociocultural positionings as well as writing memos to document and further critique and interrogate her thought processes and decisions made. The most significant challenge was contextualizing what may have been considered acceptable in 2011 but would be considered stigmatizing today. The other three interdisciplinary authors are clinicians (physician, nurse practitioner, and physician assistant) who are members of and provide care to the transgender and non-binary community.

### Analysis

De-identified, un-coded transcripts with field notes were uploaded into Atlas.ti version 8.4.4, a qualitative data management software. Initial reading of transcripts and both inductive and deductive coding was performed by the first author in 2018. Deductive or theoretical coding ensured that the preconceived research questions were answered, while inductive coding allowed for organic concepts to be identified [34]. An iterative codebook was developed with the support of two cisgender women research assistants with expertise in transgender health and qualitative methods. Transcripts were re-coded into meaning units in 2020 using conventional content analysis [29] and a structured codebook (see additional file 3) to reflect improved knowledge of qualitative methods and rigor by the first author [28, 35]. Moreover, as qualitative content analysis is reflexive and interactive, the coding system was modified over time to accommodate new insights gained [28] and an audit trail was kept to document decisions made during the analytic process. Codes were compared in an iterative process across transcripts to identify similar or contrasting beliefs, experiences, and values [36]. Common ideas among the codes were clustered into categories and then grouped into broad, overarching themes. Co-authors were provided the codebook that included definitions, exemplar quotes, and the meaning units organized into categories and themes to audit the findings and confirm the results. Subtheme descriptions and exemplar quotes were revised based on this feedback. A saturation table was created to capture the breadth of information collected [37].

### Rigor

Standard measures were taken to promote trustworthiness of the results (i.e., credibility, transferability, dependability, and confirmability) and reduce bias in the analysis [38]. It was not feasible to receive feedback on the analysis by the participants (i.e., member checking) due to the time between data collection and analysis. Therefore, three interdisciplinary clinicians that provide care to transgender people in large mid-Atlantic cities were consulted to elicit alternative interpretations and meaning of the data (i.e., peer debriefing) and increase the credibility of the results. The diverse research team (i.e., disciplines, topical expertise areas, gender identities, and communities of residence) was intentionally created to encourage and triangulate a variety of perspectives, and consequently influence the presentation of the findings. Measures intended to promote transferable results included recruiting a purposive sample and developing thick descriptions of the themes. Measures intended to promote dependable and confirmable results included keeping an audit trail (available upon request) and engaging in a reflexive practice (described above under Research team and reflexivity). Un-coded transcripts were also used with the intention to promote the rigor of a secondary analysis [39].

## Results

### Participant characteristics

Participants consisted of family medicine physicians (*n* = 5), physician assistants (*n* = 3), nurse practitioners (*n* = 2), endocrinologists (*n* = 2), and an adolescent medicine physician (*n* = 1). Their practices consisted of primary care patients at a local LGBT health center (*n* = 8), specialty care at a large academic medical center (*n* = 4), and private practice (*n* = 1). Eleven participants identified as women and two as men. The majority identified as White (*n* = 9), and the remaining identified as Black (*n* = 3) and Latina (*n* = 1). No participant identified as transgender. Ages ranged from 31 – 58 (*mean* 46.7 years) and had been providing health care to patients from 2.5 to 29 years (*mean* 17 years). Many had master’s degrees in public health (*n* = 4) or PhDs (*n* = 2) in addition to their health care degrees. A summary of participant characteristics can be found in Table 1.

**Table 1:** Participant and interview characteristics

Study ID	Age (years)	Race	Gender	Job title	Years in Practice	Interview length (minutes)
200	42	Black	Female	Physician assistant	15	^*^
207	55	White	Female	Staff physician	13	75
210	31	Black	Female	Staff physician	2.5	50
212	54	Black	Female	Physician assistant	28	65
213	31	White	Female	Nurse practitioner	3.5	75
214	58	White	Male	Physician	28	90
215	48	White	Female	Staff physician	17	75
218	33	White	Female	Staff nurse practitioner	5	48
220	52	White	Female	Chief Medical Officer	22	75
223	57	White	Female	Professor of Medicine and Oncology	29	30
224	42	White	Male	Assistant Professor of Medicine	10	50
227	57	White	Female	Assistant Professor	22	55
239	40–45	Latina	Female	Adolescent and Young Adult Medicine Division Chief	25	60

*Note*. *Information not recorded at the time of interview

### Themes

Five themes and 13 subthemes were constructed from the data. The major themes included: (1) Knowledge Acquisition: Formal and Informal Pathways to Competency; (2) Perceived Challenges and Barriers: *I didn’t know what I was doing*; (3) Power to Deny: Prescriptive Authority and Gatekeeping; (4) Stigma: *This is really strange, and I can’t really understand it*; (5) Reflections: Strategies for Success, Rewards, and Personal Motivations. An overview of the relationship between the study’s research questions, themes, subthemes, and meaning units can be found in Table 2. Four out of the five themes were identified in every interview, and all subthemes were identified by the 5th interview providing evidence that saturation was achieved (see additional file 4) The authors’ prior qualitative (KS & TP) and clinical expertise (OD, JT, RM, TP) affirmed that the depth of participants’ description of their experiences with transgender people and meaning saturation were also met. A summary of each theme is provided below.

**Table 2:** Overview of the relationship between research questions, themes, subthemes, and meaning units

Research question	Themes	Subthemes	Meaning units
(1) Attained and utilized information	1. Knowledge Acquisition: Formal and Informal Pathways to Competency	Formal pathways to competency	Healthcare school/residency exposure Organizational training
		Informal pathways to competency	Mentor Self-taught Patient
(2) Perceived barriers and facilitators and (3) Understood gaps in their professional training	2. Perceived Challenges and Barriers: “*I didn’t know what I was doing”*	*“People don’t know how to treat”*: Knowledge gaps in providing care	General knowledge gaps Scientific knowledge gaps Specific knowledge gaps Knowledge gaps that harm Knowledge gaps based on caseload
		*“She was not comfortable”*: Establishing a patient-clinician relationship	Reciprocal distrust Meeting patient expectations
(2) Perceived barriers and facilitators	3. Power to Deny: Prescriptive Authority and Gatekeeping	Gatekeeping: The stigma, discomfort and underlying denial of hormone therapy	Conditional access to hormone therapy Discomfort providing care Collegial and organizational pressure Fear of malpractice Fear of hormone permanency Fear of hormone side effects Fear of referrals
		Exceptions to the gatekeeping rule	Continuation of hormone therapy Initiation of hormone therapy Recognition of power of prescribing
(2) Perceived barriers and facilitators	4. Stigma: “*This is really strange, and I can’t really understand it”*	Negative stereotypes: “*They’re really homosexuals that are afraid to admit it”*	*“Risky sexual behaviors”* Homophobia Difficult patients
		Physical appearance: “*It’s this obsession*”	Physical appearance preoccupation Passing prejudice
		*“This is really strange, and I can’t really understand it”*	Discomfort Disbelief Discrimination
		*“Go all the way”*	Goal of invisibility
(2) Perceived barriers and facilitators	5. Reflections: Strategies for Success, Rewards, and Personal Motivations	Becoming proficient: Strategies for success	Patient-centered care Time/practice Clinical environment Confronting fears and biases Mentorship from within
		Why I provide care	Rewards and benefits Doing my job *“Tikkun olam”*

### Theme 1. Knowledge acquisition: Formal and informal pathways to competency

Knowledge about transgender health care was acquired in one of two ways: formal and informal pathways.

### Formal pathways to competency

Formal pathways to knowledge acquisition included exposure to skills or knowledge related to transgender health care, or transgender people, during residency or volunteering at a clinic. One participant stated:

I volunteered on Saturdays’cause I work during the week…I didn’t know anything about trans, anything, and it seemed like a good opportunity for me to learn and grow and challenge some of my own assumptions about gender and about who transgender people were and what it meant to be trans. (PA-200).

The majority learned through attending training at their place of employment, e.g., *we had one staff meeting where she did sort of a trans medical care 101 and she talked about hormones and surgery and labs and that kind of stuff* (PA-200). Many participants discussed exposure to skills or knowledge tangentially related to transgender care that helped bridge their learning. For example, an adolescent medicine physician already had a deep understanding of hormones, saying *you really have to know hormones to a tee* (MD-239) which increased her comfort prescribing hormone therapy. Others were exposed to sexual minority patients or topics during formal schooling, which sometimes applied to transgender people *usually there are like two lectures on GLBT issues and that’s called inclusiveness…there’s not really much medical information* (MD-220). Most participants did not have formal training about transgender health care during their education.

### Informal pathways to competency

All but one clinician discussed obtaining knowledge through informal pathways. This included learning through a combination of peer mentorship and self-instruction via experimentation or through books, conferences, online protocols (predominantly the World Professional Association for Transgender Health and the Endocrine Society guidelines), or other online searching methods. One participant reflected on their education as a self-learning process. She stated, *I’ve spent hours on self-research. I’ve read every book, all the latest books that have been written by the medical community* (PA-212). Another talked about using trial and error, *I tend to transition people over to patches, you know, over the age of 50 or 55, but there’s not a real standard for that. It just, it seems to make sense* (MD-224). Expert mentorship was important and most frequently sought within a place of employment; as this clinician said *[I go to] the person who knows the most about transgender [care]. It’s just the quickest and the easiest to go to…someone who has more experience in dealing with transgender care (MD-210)*. Absence of workplace mentorship drove some to consult external colleagues. A few discussed learning by using the patient’s experience as a guide after conferring with a mentor or online resource. One participant said *when she [my patient] came to see me, she said I prefer the injectable, and I said, great let me look it up, looks like it’s fine, you know? What’s the amount you were on before?* (PA-227). Every clinician over the age of 50, and nearly every clinician with more than ten years of experience, discussed being self-taught. Conversely, every clinician with less than ten years of experience noted mentorship as instrumental to their learning.

### Theme 2. Perceived challenges and barriers: *“I didn’t know what I was doing.”*

Challenges and barriers to care included not knowing how to care for transgender people, feeling that scientific guidance was limited, fear of providing non-standardized care, and managing multiple needs.

### *“People don’t know how to treat:”* Knowledge gaps in providing care

When recalling their first interaction with a transgender patient, clinicians consistently discussed becoming aware of their knowledge gaps, including not knowing what to do or whom to ask. Some would refer out to another clinician, specifically to initiate hormone therapy. Clinicians lacked knowledge about medications and dosing, endocrinology, the appropriate medical history, gender-affirming surgical options, social issues (e.g., how to write a medical letter to enable a patient to change a sex marker on a driver’s license), and language (e.g., pronouns, definitions). Two clinicians shared stories about times when their knowledge gaps contributed to patient harm, such as failing to consider that a transgender patient used their penis during sex, resulting in a weeks-long delay in diagnosing syphilis.

Although every nurse practitioner and physician assistant disclosed personal knowledge gaps, medical doctors usually attributed their gaps to external factors such as limited evidence or limitations in their patient panels. For example, a physician stated, *the right* [hormone] *therapy is not precisely known…there aren’t great comparative studies* (MD-224). Another explained uncertainty about who should receive treatment this way: *there are more issues of really who should be treated, and are we doing the right thing, and how do you be sure…we don’t as medicine, we just don’t understand what it really is* (MD-223). Clinicians also felt whichever gender or age group they cared for most often determined the knowledge they possessed. Consequently, they lacked knowledge about the medical needs of people in other age groups or with other gender identities. This skewing of patient panels contributed to needing more time to become knowledgeable and confident about treating diverse transgender people. When mental health or other specialized care needs arose, sourcing referrals presented a challenge due to a scarcity of knowledgeable clinicians. Clinicians noted that finding *mental health professionals that are experienced in transgender care is really difficult, especially…if the patient doesn’t have cash to kill* (MD-207).

### *“She was not comfortable”:* Establishing a patient-clinician relationship

Establishing strong patient-clinician partnerships was key to gaining proficiency in caring for transgender people. However, there were two main barriers: mutual distrust and meeting expectations. For the former, clinicians talked about patients not feeling comfortable divulging certain things such as a complete sexual history due to, e.g., *want*[ing] *to please you* [the clinician] (PA-227) or due to lack of shared identity. On the other hand, clinicians also felt patients would, e.g., *tell you what they think they need to tell you to get hormones,* (MD-220) take a higher dose of hormones, or *turn around and sell what you’re giving them because they have street value* (MD-224). These instances reaffirmed clinician distrust of patients. However, clinicians also disclosed how they perpetuated distrust, including making stigmatizing remarks such as *I don’t mean to beat a dead horse, but I’m like, are you really only having one partner? And what exactly are you doing?* (NP-218).

Conflict due to differing expectations also precluded the formation of strong partnerships. Some clinicians felt transgender patients *all want hormones, and they ask for the hormones by name* (MD-239) and want their transition *to be instantaneous* (MD-210), which conflicted with what clinicians’ thought was realistic. Dispelling the *lore about how well they* [hormones] *should work and what works best* (MD-224) and *tell*[ing] *the patient that it* [transition] *takes time* (MD-239) felt contentious to clinicians. For example, some patients preferred a particular medication based on the belief it would produce better results, while clinicians were concerned about the associated risks.

### Theme 3. Power to deny: Prescriptive authority and gatekeeping

Medical gatekeeping refers to the practice of a clinician deciding whether a patient receives the care and services they are seeking. Clinicians discussed gatekeeping and the power to make decisions – usually regarding prescribing hormones, less frequently about providing primary care. Clinicians often did not apply gatekeeping practices to patients who were already using hormone therapy. All but one clinician over the age of 50 discussed gatekeeping practices they had performed. In contrast, the majority of those under the age of 50 talked more about their position of power as a gatekeeper and efforts to reduce gatekeeping.

### Gatekeeping: The stigma, discomfort, and fear underlying denial of hormone therapy

The most frequent gatekeeping practice was requiring a letter of support from a mental health professional or proof of engagement in mental health services before prescribing hormone therapy. Additional practices included requiring a homogenous transgender narrative; smoking cessation; initiations of social transition (e.g., dressing in specific clothes); financial, emotional, and physical stability; and a commitment to undergoing surgery and achieving standard-range sex hormone levels.

Clinicians attributed gatekeeping practices to personal discomfort with the concept of a transgender person and limited treatment knowledge. Discomfort providing hormones to non-binary patients was common; that is, people whose gender identities are outside the binary of female or male. As one clinician stated:

I wouldn’t say you’re fucked up, but I would say, I don’t prescribe hormones in this situation. I’m okay setting that limit. It’s…my limit for my behavior. I guess I feel…if you don’t want to be in either gender, why do you need hormones? (MD-220).

Denial of care was justified by lack of knowledge, e.g., *if I don’t know what I’m doing, I’m not going to see this person because I won’t be able to treat him appropriately* (MD-210).

Workplace climate affected gatekeeping. One clinician reported not prescribing hormones because doing so became *very out of favor, and you couldn’t even say the word, so to speak, in this institution* (MD-223). Other clinicians described refusing treatment based either on lack of trust, or conversely, total confidence in the judgment of collaborating mental-health clinicians. For example, one clinician required patients to get mental-health care within their own institution because they found it *very difficult to trust whoever this* [unknown] *therapist was* (MD-223). Another clinician stated they *can’t undermine them* [psychologist colleagues]*. And for whatever reason, they’re telling me that I should wait, and that’s where my comfort level is* (PA-212).

Fear of personal and professional consequences perpetuated gatekeeping practices. Specifically, clinicians feared malpractice litigation based on medication side effects, irreversibility of treatments, and perceived violations of the Hippocratic oath or accepted standards of care. One clinician reported being *more concerned about testosterone* [than estrogen] *because it’s not very reversible* (MD-220). Another clinician was more concerned about estrogen, stating: *the complications. You die from it. You give someone high-dose estrogens then they’re going to have a thrombotic event* (MD-223). While several clinicians expressed fear of precipitating thrombotic events, none reported cases among their patients. Some clinicians were afraid of general harm, especially with youth: *the feeling of oh, my God, if we screw this kid up* (MD-215). Only one clinician noted the *huge impact* [of hormones] *on their* [patients’] *quality of life* (MD-224), which counteracted their concern about treatment risks.

Some clinicians expressed concern about the lack of data to support gender-affirming care, stating, e.g., *Show me the papers. Show me the data. Show me the research that says you should be doing this for patients* (MD-214). One participant felt many clinicians were concerned about their license. This participant noted that they would not provide care without on-the-job guidance, stating *I think in the end, I feel like my license is still on the line for doing something that technically I’m not quote-unquote trained to do* (MD-210). The person who trained clinicians on transgender care wielded tremendous power in how prescribing decisions were made and the workplace culture around it. One clinician recollected being berated by a colleague with a shared patient, who said, *what the hell do you think you’re doing giving this guy estrogen…I’ll make trouble for you if you persist in this* (MD-214). Some felt that gatekeeping practices, particularly the letter of support, helped quell these fears by providing medicolegal protection.

### Exceptions to the gatekeeping rule

Clinicians’ willingness to continue therapy for patients already taking hormones was much greater than their willingness to initiate hormone therapy without gatekeeping practices. One stated, *a lot of the trans patients that I’ve had come to me already on hormones. And at that point, even if I do have some discomfort, I’m sort of inclined to grandfather them in* (MD-207). Patients typically were in this category by using non-prescription hormones. Two of the three clinicians willing to initiate hormone therapy for new patients without gatekeeping were physician assistants and attributed this practice to a harm reduction approach: if they did not prescribe hormones, the patient would obtain them through risker methods.

### Power to prescribe or deny

About half of the participants reflected on the power to prescribe or deny hormone therapy, including variations in how the standards of care were interpreted. For example, one stated, *some people feel like the mental health requirement is a requirement, and some people feel like it’s a suggestion…so while there are standards, I think they’re interpretable standards* (PA-200). Several clinicians talked about making decisions to prescribe based on whether it was right for the patient rather than on clinical guidelines.

### Theme 4. Stigma: *“This is really strange, and I can’t really understand it.”*

Only three clinicians did not describe stigmatizing beliefs or make stigmatizing statements, indicating that patient-clinician relationships were primarily characterized by stigma. Four subthemes arose around stigma: negative stereotypes about transgender people, transgender people’s concern with physical appearance, discomfort and discriminatory practices towards transgender people, and stigma towards people with nonbinary identities or expression.

### Negative stereotypes: *“They’re really homosexuals that are afraid to admit it.”*

Clinicians demonstrated three central stereotypes about transgender people: sexually promiscuous, closeted homosexuals, and difficult patients. Clinicians discussed concerns about sexual practices that put transgender people at higher emotional and physical risk with stigmatizing language such as “sexually promiscuous.” One clinician asserted that transgender people perform in drag shows for attention, stating, *I think it’s being accepted in that group of people who are observing them…and they’re looking for it in the wrong places obviously* (MD-212). A few clinicians conflated gender identity and sexual orientation and talked about transgender people being *homosexuals that are really afraid to admit it* (MD-223). A clinician referenced their transgender patients as *difficult patients, particularly trans women who have had pretty rough experiences, and are pretty rough people…*[with] *a higher incidence of personality disorders* (MD-220). Clinicians frequently made negative generalizations about transgender people as patients, like they are *a little more scattered* (MD-210), *kind of crazy* (MD-214), or *they can be difficult patients* (MD-220) that are *a lot harder to deal with* (MD-207).

### Physical appearance: *“It’s this obsession.”*

Clinicians made negative comments about transgender women’s physical features, such as they are *always so awkward…they don’t fully pass* [i.e., are not always perceived as a cisgender person]*…*or *they never quite get the mannerisms of women, and it always looks artificial. You can spot them a mile away* (MD-223). At the same time, clinicians were judgmental of the women’s relationship with their physical appearances, expressing sentiments like *it’s this obsession like it’s never going to be okay* (MD-207), and as a *psychological problem…*[that] *raises this flag in me* (MD-207). Only one clinician talked about the difference that economic privilege, specifically access to expensive surgeries, can make for a transgender woman who wishes to change her appearance. The notion of obsession with appearance was never discussed in the context of transgender men. Providers felt that transgender men’s physical appearances were *“generally much better, more believable”* (MD-214), even *“scary how good they…completely pass”* (MD-223).

### “This is really strange, and I can’t really understand it.”

Clinicians commonly expressed disbelief that a transgender person could ever find happiness, as exemplified by the remark, *I just looked at him and felt to myself, “Are you really happier?”…I don’t know what the answer is. Sometimes I feel like saying to somebody, “Can’t you grow out of this a little bit?” (MD-223).* Yet another clinician talked about having to convince colleagues that transgender people were real people with authentic identities. Mistreatment of transgender people based on underlying stigmatizing beliefs was only discussed as something witnessed, not practiced. Mistreatment included seeing *a lot of laughing and snickering* [at transgender patients], *and people would walk in just to see them like it was a sideshow type of thing. The doctors would flip a coin over who had to take them* [a transgender patient] (PA-212).

### “Go all the way.”

Clinicians believed that a transgender person’s goal should be to become invisible as a transgender person. For example, one clinician noted, *if you want to transition, go all the way and transition…the best thing for a trans person is to transition as fully as possible as soon as possible* (MD-220). One clinician expressed disapproval of transgender people *choosing a name that doesn’t really necessarily go in one direction or the other* (NP-218). In addition, clinicians expressed a lack of understanding of non-binary identities, exemplified by statements such as *it’s often a stage…*[and] *it’s a social statement, and you don’t treat things with medications that are social statements* (MD-220).

### Theme 5. Reflections: Strategies for success, rewards, and personal motivations

Every clinician discussed: strategies that increased their proficiency in caring for transgender people; reflections on how their ideologies, identities, and background impacted the care they provided; or sentiments about why they provided care and how they benefited from caring for this patient population.

### Becoming proficient: Strategies for success

Clinicians shared several individual and structural strategies for building trusting patient-clinician partnerships. Strategies included attempting to reduce hierarchical power dynamics through partnering with patients by *do*[ing] *things together step by step* (MD-214) and creating a safe space by initiating the visit with *what their* [the patients] *goals are. What they would like to accomplish* (PA-212). Some discussed the importance of using time, especially during the extended initial visit, to listen to patients and *learn a little bit about what these people are facing* (MD-224) in other aspects of their life, i.e., [to] *focus on the patient as a human being, as a person* (MD-239). Yet another tactic was being humble and transparent, such as telling patients *I don’t have any standardized basis for how to do this, but I will be happy to work with you* (MD-214).

Clinicians also talked about the importance of communicating honestly, realistically, and prioritizing the patient’s goals. *Tell*[ing] *them* [patients] *basically what they can expect* (PA-212) was an important tool that experienced clinicians did to align their patient’s expectations with what hormone therapy could produce. Experience also contributed to improved communication and a better understanding of the barriers and concerns around accessing gender-affirming care as one clinician learned: *very few people persist in saying they don’t* [want gender-affirming surgery] *when you figure out how to ask the question right, as in if it was free and safe and you weren’t afraid of general anesthesia…It’s a good protective mechanism to say you don’t want something you can’t have* (MD-220).

Some clinicians described the need to engage in self-reflexive processes to overcome barriers in developing proficiency such as to *face their own fears and get comfortable treating different types of people that they had not seen in their training* (PA-227). Many talked about experiences of initial discomfort or anxiety when caring for a transgender or nonbinary patient, related to not knowing *what am I supposed to do?* (PA-212), being *so fearful of failure* (MD-239) and not *doing it right* (MD-220). One clinician talked extensively about *the goals of therapy as sort of fundamentally different than some of the other things that we do…instead of looking at lab tests to be sure their hemoglobin A1C is better…you’re asking them how they feel* (MD-224). Empowering patients to drive medication dosing based on their own risk tolerance signaled an important shift from clinician-driven care to person-centered care. Some clinicians acknowledged the need to *look at your own biases, what you bring to the table* (MD-239) to provide care successfully. This included the need to resist the *societal push to be a beautiful woman* (PA-200) when that was not the patient’s goal and the prejudice of over-testing transgender patients for sexually transmitted infections due to *assuming that their behavior is riskier* (NP-218).

Structural strategies included curating a workplace environment that facilitated supportive and inclusive care. This included hiring a diverse and culturally competent staff including transgender people; promoting a nonjudgmental culture; reducing restrictions on medications and limitations on visit durations; and putting systems in place to support patient care, such as electronic medical record templates for clinicians to easily write a letter of support for a patient to obtain a passport or driver’s license. Mentors were important for providing resources, supporting less experienced clinicians, and reviewing medical notes to ensure they had *asked the right questions* (NP-218). Mentorship and experience were felt to increase clinician comfort more than formal training or certification in transgender care. Lastly, having a knowledgeable and trustworthy referral system, such as the ability to *work very closely with the psych clinic here* (MD-223), was crucial in removing barriers for both the clinician and patient.

### Why I provide care

Most clinicians organically discussed their motivations and the benefits of providing transgender care without being prompted. Although a few perceived transgender people as interesting or *fringy, on the edge alternativeness* (MD-215), most were motivated to provide care by a desire and commitment to *those who can’t just go anywhere and get healthcare, who have problems or barriers in healthcare* (PA-212). Two clinicians told a story of being changed after their first experience with a transgender patient: *whatever views I may have had or never had…all I see in front of me is someone who is sick, and I just need to treat them* (MD-210). Other reflections included the satisfaction that came after building a trusting relationship with a patient, the joy of *seeing the transition of how the person changes, like how they’re happier* (MD-210) after they transition, and the knowledge that they supported a patient to *achieve in most cases a lifetime goal for themselves* (PA-212). One clinician talked about the rewards as *one of those things where you feel like it’s mutual* (PA-227). Another spoke about it increasing their compassion and desire *to promote equality and rights* (NP-218) for the greater transgender community.

## Discussion

### Stigma, gatekeeping, knowledge deficits

Our results support prior literature that stigmatizing beliefs, gatekeeping practices, and knowledge deficits are common in transgender health care [2, 3, 5, 18]. However, this study extends the discussion by deepening our understanding of clinicians’ perspective on these issues, and how they grapple with them to fulfil their desire to provide care to this patient population. For example, the desire to provide high-quality care alongside pervasive and stigmatizing perceptions about transgender people was striking throughout the interviews. Most clinicians felt that caring for transgender people was rewarding. At the same time, they held strong biases about patient identities, practices, and priorities. Stigmatizing and essentializing stereotypes—from assumptions that only a singular transgender narrative exists to beliefs that having multiple partners or being “homosexual” is inherently wrong—perpetuated negative beliefs about transgender people, negatively impacting patient-clinician interactions. For example, clinicians’ paradoxical criticism of transgender women for obsessing about their appearance while simultaneously not being believable as women illuminates the pressure transgender women face for their physical appearance as well as the dilemma they meet when their clinicians also partake in applying this pressure. At the same time, clinicians felt it was scary how transgender men can be perceived as men, suggesting that the ability to blend in with cisgender men or not easily be identified as transgender should be feared. These views reinforce the transphobic idea that transgender women are not really women and transgender men are not really men. This creates an untenable position for the transgender person: to be seen as transgender increases vulnerability to violence [2, 40, 41], but to be seen as cisgender inspires fears in cisgender people that transgender people are tricksters, “malicious in their deception.” [42] When discussing maladaptive choices or behaviors, few clinicians acknowledged that these were not a result of being transgender, but rather a consequence of persistent rejection and harm from family members, jobs, neighbors, religious communities, clinicians, and society in general [2]. One might have expected clinicians who did not express stigmatizing beliefs to express positive feelings about providing care to transgender people. Interestingly, the clinicians who expressed stigmatizing thoughts simultaneously endorsed positive sentiments on providing care for transgender people (see additional file 4). This demonstrates the complex, unrelenting, and insidious nature of stigma that is perpetuated from interpersonal interactions to structural level diagnoses, policies, and laws [43]; implicit bias may also be playing a role in this association but was not measured in this study [44].

This study provides important insight into the clinician’s thought process behind gatekeeping, which was influenced by both stigma and knowledge deficits. Clinicians broadly understood gender as a binary and pathologized those with nonbinary or genderqueer identities or expressions. Some would not initiate hormone therapy or prescribe gender-affirming hormone therapy to nonbinary people. Hormone therapy is an essential treatment for nearly 50% of non-binary adults [2]. Seeking a clinician who is willing to prescribe hormones is common for binary and nonbinary transgender youth [45]. Moreover, literature shows gendered differences in healthcare experiences within gender-diverse populations [46], including that nonbinary people may have poorer health outcomes than binary transgender women or men [2, 45, 47]. Gatekeeping, particularly by physicians, was justified by citing the complications and permanency of hormone therapy, litigation risk, or not having the knowledge or anyone to ask. Clinicians’ aversion to risk appeared to supersede a patient’s tolerance to risk. Clinicians are trained to understand the potential positive and negative effects as well as the safety of medications and communicate these to patients when discussing if a particular therapy may be right for them. However, for gender-affirming hormone therapy, the clinicians’ concern for safety and undesired effects far outweighed consideration of potential benefits. Recent data support that gender affirming therapies can dramatically improve quality of life and can be lifesaving [48, 49]. However, only one clinician providing this care spoke of perceiving potential benefits, while a great majority discussed their concerns for risks. Clinicians also did not discuss the risks of not prescribing gender-affirming therapies, among which may be worse general and mental health, increased suicidality, increased vulnerability to transphobic violence, and the permanent effects endogenous hormones have on an adolescent’s development [8, 50, 51]. However, some clinicians did note that gatekeeping may result in people attempting to treat themselves, such as buying non-prescribed hormones on the internet, increasing risks for serious complications [52, 53]. Neither discomfort nor fear stopped participating clinicians from providing care to transgender people, which was a prerequisite for study participation. However, they did appear to inform the type and quality of care provided. Our findings support literature [8, 54] that demonstrates gatekeeping perpetuates a harmful cycle in which patients fear being honest with clinicians in anticipation of being denied gender-affirming care which then [8, 54]reaffirms clinicians’ distrust of transgender people. Clinicians did not seem to connect patients’ fear of honesty as directly related to their gatekeeping. This cycle of behavior may contribute to clinicians missing opportunities to expand their knowledge, reinforcing incorrect beliefs about what it means to be transgender. Clinicians did not mention the historical medical mistreatment of transgender people—pathologization, harm, and discrimination [2, 55]—when discussing the challenges establishing patient-clinician relationships.

### Strategies for successful care delivery

Our results also move beyond the current knowledge base to illuminate factors that contribute to providers’ successful delivery of care to transgender people. Online clinical guidelines, continuing education at conferences, and expert mentorship were important ways of learning how to provide care as well as increasing comfort to provider care for those who didn’t have formal education in transgender health topics. On-site mentorship is a particularly important facilitator to increase comfort in providing transgender care among early-career clinicians, particularly since discomfort promoted gatekeeping and refusal to provide care. Moreover, clinical experience increased comfort more so than formal training or certification, additionally pointing to the importance of mentorship when encountering patients in the clinical setting. Beyond gaining practical experience, a key theme for becoming proficient in providing transgender health care was building trusting partnerships with patients. Strategies included creating a safe space and time in the initial visit to hear a patient’s story and concerns, focusing on the patients goals versus achieving a specific blood level or transition destination, providing anticipatory guidance and setting realistic expectations about hormone therapy, being humble about lack of experience or knowledge gaps, sharing decision-making about risk tolerance, and engaging in a reflexivity practice that allows self-reflection and critical examination of one's biases and prejudices. These strategies can be summarized as engaging in a person-centered and harm-reduction approach to care.

A person-centered approach, in which clinicians share power with the patient to direct their treatment, becomes critical for healing the relationship between patients and clinicians, promoting gender-affirming experiences, and better care outcomes [56, 57]. This also allows for clinicians’ understanding of gender to evolve, as the approach creates room for clinicians to learn about the expansiveness of gender. Additionally, an informed consent model, well-established in hormone therapy, acknowledges that people have the right to accept risks in medical treatment [58, 59]. There is an important role for the integration of harm reduction models, which aim to prevent harm and center patients’ goals and preferences, especially those who are marginalized, with poor access to and distrust of healthcare professionals [60, 61]. This requires the clinician to understand that the patient is seeking to have their needs met; if those needs cannot be met through the healthcare system, the person will attempt to care for themselves with the resources they have and may be harmed in this process [61].

Lastly, structural-level supports were also important to reduce barriers and challenges to providing care to transgender people. This included organizations that fostered a clinical environment of inclusivity and provided competency training for clinicians and staff alike, supported longer clinical visits to allow for a trust to be developed, and provided resources like templates for writing letters to social services, referrals who were vetted for transgender competency, and case managers that coordinated other social and care needs for the patient. It’s important we emphasize clinicians capacity and willingness to provide care to transgender people is not just an individual issue, but something that is inextricably intertwined with societal norms, systems, and institutional policies that continually reproduce stigmatization and constrain access to resources [11]. It will be insufficient to only improve educational curricula or teach person-centered care without examining ways institutions, organizations, and systems support and uphold ways of operating that perpetuate harm and center only the experiences of the privileged.

### Healthcare practice and education recommendations

The insidious, stigmatizing beliefs and a failure to situate transgender peoples’ experiences within the larger socio-ecological context of their lives demonstrated that increased medical and surgical knowledge alone will not be sufficient to overcome harmful care experiences and access to care barriers. Clinicians need to be supported in examining how they themselves may enact harm. Our results demonstrate that healthcare and continuing education must be comprehensive and include a more nuanced understanding of the diversity of gender identity and expression; the history of medical trauma, including misuse of clinicians’ power to deny care and harm patients as well as ongoing healthcare aggression enacted against transgender people [62]; current barriers to care; cultural humility and the role of implicit bias in perpetuating health disparities; and a holistic view of transgender care needs including social and financial needs such as such as writing a medical letter to enable a patient to change their legal sex marker or engaging in social actions to protect transgender people from violence. The more clinicians can become aware of their biases and learn to embrace and respect differences, the less stigma will dominate and influence the care provided to all people [63, 64].

Education should also move beyond lectures and include clinical simulation exercises to practice effective, respectful communication [65, 66]. Incorporating experiential learning into the standard curriculum may be a way to increase clinician comfort [67] as well as normalize communication around other topics that are also often stigmatized, such as taking an inclusive sexual history, broaching topics like sex work, and motivational interviewing to promote smoking cessation. Within healthcare schools, education should target faculty as well as students to foster role models and increase capacity to care and educate in all contexts, from pediatric to reproductive health care [15]. The development of validated tools to assess gaps in knowledge and biases among learners is warranted to continually identify deficiencies in educational initiatives [15]. Educational settings also have a responsibility to support transgender learners, faculty, and staff; eliminating stigma toward transgender people requires that healthcare training programs embody inclusivity of transgender people’s identities, lives, and needs, which is currently lacking [19, 68, 69]. Although the data in this study are older, recent findings from a diverse sample of transgender community members corroborate the ongoing relevance of our themes and educational recommendations: (1) increased comfort with transgender patients; (2) shared medical decision-making; (3) reduced stigmatizing assumptions; (3) increased knowledge of sexual behaviors and transgender health; (4) increased knowledge of health impact of social determinants of health [70].

### Organizational and policy recommendations

Institutions should invest in recruiting professionals with expertise in caring for transgender people and mentorship development programs, an often-overlooked area, and every staff member who interacts with patients should be trained to provide inclusive services, no matter their role. Case managers should be recognized as an essential part of the care team, skilled in addressing social factors that impact patients’ health [56, 71]. Healthcare professional organizations can support clinicians by issuing position statements, recommendations, policies, and clinical guidelines that promote scientific and evidence-based care for transgender people and may assuage some of the fear of malpractice that prevented clinicians in this study from providing care. Policies at the local, state, and federal levels must also protect transgender people’s access to healthcare as critical factor in shaping health outcomes [5]. This is particularly relevant as over half of US states have considered legislation banning best-practice medical care for transgender youth in 2020 and 2021, and Arkansas became the first state to pass a ban into law in April 2021 [72].

### Limitations

As with any healthcare field, transgender standards of care change over time as medical knowledge advances; therefore, what may indicate lack of knowledge today, may have been best practice ten years ago. The findings of this study should be situated within the context that the Standards of Care V6 were in use the year this study was conducted, and V8 is anticipated to be released this year (2022). There has also been a growing public awareness of nonbinary as well as genderqueer identities over the years. In this study, nonbinary and genderqueer identities are included under the transgender umbrella. However, we recognize that this is nuanced, and not every nonbinary or genderqueer person considers themselves to be transgender.

The small, cis, majority-white, mostly female sample recruited from US-based settings known to provide care to transgender people limits the transferability and generalizability of the results. It’s noteworthy that none of the clinicians in this study identified as transgender or nonbinary, since transgender clinicians are also transgender patients and therefore view transgender health care from both sides of the examination table, as it were. There is no universal transgender experience, but we might expect that findings—such as knowledge of transgender care, pervasive stigma, difficulty with patient-clinician trust-building, and clinicians’ fear of hormones—may be mitigated when a clinician is a person of transgender experience. Providing care may carry unique motivations and personal fulfillment, as well as unique barriers, for transgender providers. Further research that is inclusive of a more diverse sample of clinicians, working in a variety of clinical settings and countries, with a range of experience providing gender-affirming care is needed to explore these possibilities.

As with all secondary data analysis, the changes in social, cultural, and political norms from the time the data was collected until analysis may have led to misinterpretations [39]. To address this, some terminology that may have been still acceptable in 2011—such as “transgenders,” “transsexuals,” or “biologically female”—were not coded as stigmatizing. The inability to validate the findings among the participants or triangulate the transcripts with other types of data limits the credibility of the results; however, peer debriefing was pursued by confirming the results with clinicians that could represent the participants*.* Moreover, additional file 4 demonstrates that data saturation was achieved after the fifth participant despite the nature of this secondary analysis. Thus, there was more than enough data and engagement with participants to develop thick descriptions in response to the research question.

Finally, all the researchers involved in data collection identify as cisgender women which is both a limitation and benefit to the credibility of the results. The entirely cisgender sample may have felt more comfortable disclosing certain practices and attitudes to a peer than someone who identified as transgender and a non-clinician. The primary coder was able to bring a fresh, etic perspective to clinical challenges as she is not a clinician herself, and the other co-authors brought emic or insider perspectives, balancing out biases in the analysis and presentation of results.

## Conclusions

This study contributes to and directly responds to three priorities identified as critical in transgender health disparities research: (1) determining gaps in knowledge among clinicians; (2) determining indirect barriers (e.g., stigma, environment); and (3) identifying potential solutions to overcome barriers to providing transgender health care [4]. Previous literature on barriers to health care for transgender communities has focused on the patient or student perspective and does not explain motivations for providing care or factors that interfere with clinicians’ willingness to deliver care [73–75]. This study sheds light on clinicians’ feelings about providing care to transgender people, factors that motivate behaviors such as gatekeeping, and ways clinicians become adept and challenges they face in providing gender-affirming care. Although it is likely that early-career clinicians now have more exposure during formal schooling than they did decades ago, our data suggests that clinicians need more than formal education, and recent data corroborates there are still looming gaps in curriculum and persistent biases against transgender people despite increased knowledge [10, 18]. We strongly urge more research be conducted on positive assets of care and stigma among interdisciplinary clinicians to understand how the past decade of increased education, training, and guidelines has impacted care experiences. The insights into the clinician experience presented in this paper should complement advances in curriculum, training, and structural improvements, as well as serve as a roadmap to addressing clinician factors that contribute to health care access barriers.

## Supplementary Information

**Additional file 1.** 

**Additional file 2.** 

**Additional file 3.** 

**Additional file 4.** 

## Data Availability

All relevant data analyzed during this study are included in this published article and its Supplementary Information files.
