# Supplementary material for: Interdisciplinary clinicians’ attitudes, challenges, and success strategies in providing care to transgender people: a qualitative descriptive study
Source: BMC Health Serv Res. 2022 Sep 8;22:1134. doi: 10.1186/s12913-022-08517-x (PMC9454229; doi:10.1186/s12913-022-08517-x)
Supplement: Supplementary file 1 — Additional file 1. [file 12913_2022_8517_MOESM1_ESM.docx]

Consolidate criteria for reporting qualitative studies (COREQ) checklist

| **Item** | **Description** | **Application to Study** |
| --- | --- | --- |
| **Domain 1: Research team and reflexivity** | | |
| Personal Characteristics |  |  |
| 1. Interviewer/facilitator | Which author/s conducted the interview or focus group? | Senior author |
| 2. Credentials | What were the researcher’s credentials? | Senior author: PA, MPH; now Ph.D.  First author: MSN, RN, Ph.D. candidate |
| 3. Occupation | What was their occupation at the time of the study? | Senior author & first author: Ph.D. candidates (at time of interview and analysis, respectively) |
| 4. Gender | Was the researcher male or female? | Both: Cisgender females |
| 5. Experience and training | What experience or training did the researcher have? | Both: Qualitative methods training in Ph.D. program |
| Relationship with participants |  |  |
| 6. Relationship established | Was a relationship established prior to study commencement? | Six of the participants were known to the senior author |
| 7. Participant knowledge of  interviewer | What did the participants know about the researcher? | Participants were informed about the aim of the research |
| 8. Interviewer characteristics | What characteristics were reported about the interviewer/facilitator? | Interest in and purpose for research included in reflexivity statement |
| **Domain 2: Study design** | | |
| Theoretical framework |  |  |
| 9. Methodological orientation and theory | What methodological orientation was stated to underpin the study? | Qualitative descriptive methodology |
| Participant selection |  |  |
| 10. Sampling | How were participants selected? | Purposive |
| 11. Method of approach | How were participants approached? | Email |
| 12. Sample size | How many participants were in the study? | 13 |
| 13. Non-participation | How many people refused to participate or dropped out? Reasons? | None |
| Setting |  |  |
| 14. Setting of data collection | Where was the data collected? | Project office, clinicians’ offices or home, car, restaurant, and telephone |
| 15. Presence of non-participants | Was anyone else present besides the participants and researchers? | No |
| 16. Description of sample | What are the important characteristics of the sample? | Majority female, cis, White, highly educated, older, and were seasoned clinicians |
| Data Collection |  |  |
| 17. Interview guide | Were questions, prompts, guides provided by the authors? Was it pilot-tested? | An interview guide was followed |
| 18. Repeat interview | Were repeat interviews carried out? If yes, how many? | No |
| 19. Audio/visual recording | Did the research use audio or visual recording to collect the data? | Audio recorded then transcribed |
| 21. Field notes | Were field notes made during and/or after the interview or focus group? | Fieldnotes were made immediately after the interview |
| 22. Duration | What was the duration of the interviews or focus group? | 30 – 90 minutes (*mean* 62 minutes) |
| 23. Data saturation | Was data saturation discussed? | Saturation tables were made for the secondary analysis |
| 24. Transcripts returned | Were transcripts returned to participants for comment and/or correction? | No |
| **Domain 3: Analysis and Findings** | | |
| Data analysis |  |  |
| 24. Number of data coders | How many data coders coded the data? | Only the first author |
| 25. Description of the coding tree | Did the authors provide a description of the coding tree? | Included in the saturation table |
| 26. Derivation of themes | Were themes identified in advance or derived from the data? | Inductively derived codes from data |
| 27. Software | What software, if applicable, was used to manage the data? | Atlas.ti version 8.4.4 |
| 28. Participant checking | Did participants provide feedback on the findings? | Some member checking occurred in the interviews; returning findings to the participants was not feasible, but three interdisciplinary clinicians were consulted to confirm the validity of the results. |
| Reporting |  |  |
| 29. Quotations presented | Were participant quotations presented to illustrate the themes / findings? Was each quotation identified? | Participant ID was provided for quotes and can be cross-referenced with participant characteristics in Table 1 |
| 30. Data and findings consistent | Was there consistency between the data presented and the findings? | Findings provide context for and consistency of the results |
| 31. Clarity of major themes | Were major themes clearly presented in the findings? | Five major themes are included in the results |
| 32. Clarity of minor themes | Is there a description of diverse cases or discussion of minor themes? | 13 minor themes or subthemes are included in the results |
