# Supplementary material for: Interdisciplinary clinicians’ attitudes, challenges, and success strategies in providing care to transgender people: a qualitative descriptive study
Source: BMC Health Serv Res. 2022 Sep 8;22:1134. doi: 10.1186/s12913-022-08517-x (PMC9454229; doi:10.1186/s12913-022-08517-x)
Supplement: Supplementary file 2 — Additional file 2. [file 12913_2022_8517_MOESM2_ESM.pdf]

Gender and Health: Understanding Access and Risk for Transgender Adults

PI: Danielle German, IRB00003147

INTERVIEW GUIDE

Health staff

1. **Personal Background** (eg. "Tell me about yourself")
   The intent of this topic is to set the tone for the conversational nature of the interview and to get a sense of the respondent and what s/he feels is important to share about himself or herself.
   - Education and training
   - Career trajectory: when, where, why, how?
   - Current job/role: describe – what, when, how, why? Likes/dislikes.
   - Previous jobs/roles both at the organization and before arrival
   - Future goals for self at organization and beyond

2. **Organization background** (eg. "Tell me about the health center")
   The intent of this topic is to better understand the organizational structure, context, and general practices.
   - If in leadership role:
     - When organization got started, why & how, by whom.
     - How it has changed over time?
     - Future plans/goals for the organization.
   - What is your role at the health center?
     - Describe a typical day. . . .
     - How did you find out about a job here? Hiring process?
     - How long, responsibilities, changes over time
     - Why this work, in particular
     - Rewards and Challenges: pay, benefits, hours, time off, etc.
     - How do others in your role/your title experience work
     - Trans employees?
   - Norms of the health center
     - Describe organization culture
       - Probe: strict, relaxed, high pressure, open, family-like, busy
     - How is the organization structured: hierarchy/egalitarian
     - How do staff communicate with each other/leadership
     - How do policies/procedures get implemented?
     - When/how does training take place?

3. **Health services** (eg. "Tell me about services at the health center")
   The intent of this topic is to understand how patients know about and access health services.
   - What services are available?
     - Primary care, specialty care, mental health, case management, etc.
     - Who access this care? What type of patients do you see?
       - Insurance status, age, race, SES, special populations?
   - How do patients find out about this place? Why do you think they come?
     - Pros/Cons
     - Facilitators/Barriers to care
   - Walk me through a typical patient experience here – from entry to departure?
   - Describe your interactions with trans clients/patients
     - First, most recent, most memorable, best/worst, typcial
   - Interactions/friendships/relationships with trans people outside of work?

   For Clinicians (RN, PA, NP, MD, DO):
   - What is "transgender health care?"
     - How does transgender health differ from general patient population
   - How s/he learned about the practice transgender medical care
     - Where did you look for information? What did you find?
     - What did you do with this information? (how incorporate)
   - Gaps in knowledge both personally and in the field in general
   - Experience providing care to transgender patients/clients
     - First clinical/professional encounter with a transgender person
     - Narrate a typical visit or series of visits
     - How has the experience changed over time
     - Most memorable, most satisfying, least satisfying
     - Likes/Dislikes; Hard/Easy about working with this population
   - Ideal set of resources for learning about transgender care
   - Facilitators of health care access and utilization by transgender patients
   - Barriers to health care access and utilization by transgender patients
   - How does stigma impact transgender clients/patients?
   - How does HIV impact transgender clients/patients?
   -
4. **Demographics:** Age, race, gender identity, organization location, job title
