# Supplementary material for: Interdisciplinary clinicians’ attitudes, challenges, and success strategies in providing care to transgender people: a qualitative descriptive study
Source: BMC Health Serv Res. 2022 Sep 8;22:1134. doi: 10.1186/s12913-022-08517-x (PMC9454229; doi:10.1186/s12913-022-08517-x)
Supplement: Supplementary file 3 — Additional file 3. [file 12913_2022_8517_MOESM3_ESM.docx]

**Codebook and exemplar quotes**

| **Meaning Units** | **Condensed meaning units** | | **Exemplar Quotes** |  |
| --- | --- | --- | --- | --- |
| **Theme 1:** **Knowledge acquisition: Formal and informal pathways to competency** | | | |  |
| **1.1 Formal pathways to competency** | | | |  |
| Healthcare school/residency exposure | Exposure to skills or knowledge related to transgender healthcare or to transgender individuals during formal healthcare schooling, residency, or other training (e.g., volunteering) | “So they had one Saturday a month was transgender health and education, The, <laughs> and I volunteered on Saturdays ‘cause I work during the week. And yeah, I wanted to learn more about it. I didn’t know anything about trans anything <laughs> and it seemed like a good opportunity for me to learn and grow and to challenge some of my own assumptions about gender and about who transgender people were and what it meant to be trans. So yeah, it seemed like a growth opportunity. Yeah... I think just showing up to volunteer and they did a really good job with the volunteers of matching us with a person who was doing the care so they had employees who provided the care and so I got to do a lot of observing and asking questions and talking. A lot of it was around the medical stuff, so how to do hormone management, what kind of labs to follow, what kind of things to look for, what kind of things to ask in the physical exam, and things like that.” (PA-200) | |  |
|  |  |  |  |  |
|  | Exposure to skills or knowledge tangentially related to transgender healthcare or individuals during formal healthcare schooling, residency, or other training (e.g., volunteering) | Interviewer: “And is doing all of that medication management something that you learned during your residency and your fellowship?” Respondent: “Yes, I did. Uh-huh, uh-huh. And, again, as an adolescent medicine doctor, you really need to know hormones to the tee. You have to know exactly what each pill does, what it is, what are the doses and stuff like that.” (MD-239) | |  |
|  |  |  |  |  |
|  |  |  |  |  |
|  |  |  |  |  |
|  | Lack of exposure to skills or knowledge related to transgender healthcare or to transgender individuals during formal healthcare schooling, residency, or other training (e.g., volunteering) | Interviewer: “When you were in school and doing your training in medical school and stuff like that, was there ever any sort of mention of transgender?” Respondent: “Nothing.”  Interviewer: “Nothing?” Respondent: “No.” (MD-215) | |  |
|  |  |  |  |  |
|  |  |  |  |  |
| Organizational training | Transgender competency and/or organized health care training offered or required for providers through their organization | “I think the first day of orientation the chief medical officer does a sort of LGBT 101. So it’s an hour and it covers lesbian, gay, I don’t think there’s any bisexual anything, and trans, sort of language, terms, but not anything specifically in terms of medical, so it’s more of a customer service <laughs> for LGBT people. That’s how I recall it. And then I’ve been there two and a half years and we had one staff meeting where she also did sort of a trans medical care 101 and she talked about hormones and surgery and labs and that kind of stuff. That was about a year ago. But there’s nothing on an ongoing regular basis...There are protocols that the chief medical officer, that she wrote for the medical providers but they’re very medical. Visit number one, this should be the questions you ask for the history, this is what the physical exam should entail, these are the labs that you should draw. Visit two, these are the parameters, this is what you prescribe, so it’s very medical.” (PA-200) | |  |
|  |  |  |  |  |
|  |  |  |  |  |
|  |  |  |  |  |
|  |  |  |  |  |
| **1.2 Informal pathways to competency** | | | |  |
| Mentor | Transgender care learned through an experienced mentor | “To the head, the person who knows the most about transgender. That’s pretty much who I go to. I think it’s just the easiest and the quickest to go to because to be honest with you I don’t know, I can’t <inaudible> they have something they say but I still don’t think it’s enough. So you just have to go to someone who has more experience in dealing with transgender care and I know it’s probably good for me to read other things but in terms of resources I’m not sure of too many resources.” (MD-210) | |  |
|  |  |  |  |  |
|  |  |  |  |  |
|  |  |  |  |  |
|  |  |  |  |  |
|  | Transgender care learned through peer mentoring | “I've talked, actually a lot, to mid-level providers, especially nurse practitioners, who have one trans patient and need to know what to do. And I send them our protocols, although I have to read the new UCSF protocols and depending on what they are maybe I'll refer to them instead. But I have sent all our own protocols and handouts and stuff to a lot of different people.” (MD-220) | |  |
|  |  |  |  |  |
|  |  |  |  |  |
| Self-Taught | Transgender care learned through experimentation | “There’s a question on whether or not you can avoid some of these-- the hypercoagulability aspect [of estrogen therapy] by giving patches, and so typically I’ve been generally older, since the risk of cardiovascular disease and probably clots, of course, increase with increasing exposure. That is age. I tend to transition people over to patches, you know, over the age of 50 or 55, but there’s not real standard for that. it just-- it seems to make sense.” (MD-224) | |  |
|  |  |  |  |  |
|  |  |  |  |  |
|  |  |  |  |  |
|  |  |  |  |  |
|  | Transgender care learned through self-teaching (e.g., books, online protocols) | “Well, I’ve spent hours on self-research. I’ve read every book, all the latest books, that have been written by the medical community.” (PA-212) | |  |
|  |  |  |  |  |
|  |  |  |  |  |
|  |  |  |  |  |
| Patient | Patients teach clinicians about transgender health care | “I prescribe them [hormones], she’s [the patient] told me exactly what she was on when she came here, and this is exactly what I take, so then I prescribed them and then I looked it up, both on up to date and talked with one of my colleague friends who’s this gynecologist and just ran it by and said this is what I’m doing and she goes, yeah that’s fine, you know...I felt like I could probably do it just about as well as anybody else should if there wasn’t a transgender clinic with expertise someplace and I felt like I had the compassion to do it and then I could use those up to date and talking with other people to be certain that I was on target; and I was and then interactions with HIV retrovirals, and so when she came in to see me, she said I prefer the injectable, and I said, great let me look it up. Look like it’s fine. You know, what’s the amount you were on before...So and you know, maybe I’m wrong, maybe there’d be someplace else where somebody has a lot more experience in doing this, I just didn't know where.” (PA-227) | |  |
|  |  |  |  |  |
| **Theme 2: Perceived challenges and barriers: I didn’t know what I was doing** | | | |  |
| **2.1 People don’t know how to treat: Knowledge gaps in providing care** | | | |  |
| “People don't know how to treat, and they don't know if they should treat”: General knowledge gaps | Clinician knowledge gaps in providing transgender health care generally | “People don't know how to treat, and they don't know if they should treat. Some will treat, but they don't know how to.” (MD-223)  “I had concerns about-- I know the drugs, I know endocrinology, but I didn't know what I was doing.” (MD-214) | |  |
| “Who should be treated, are we doing the right thing, and how do you be sure”: Scientific knowledge gaps | Scientific knowledge needed to provide transgender health care | “The other issue is that the right therapy is not precisely known, and it’s very provincial in terms of-- in terms of the-- and in terms of what therapies are used by a given practitioner. You know, they trained with someone who knew something about transgender patients, and that’s what they have used, and that’s what they always use, and so there’s not-- there aren’t great comparative studies.” (MD-224)  “Physicians want to know how to treat these patients. They want to know what's safe and what could be done. So there's issues of treatment, but maybe there's more issues of really who should be treated, and are we doing the right thing, and how do you be sure.” (MD-223) | |  |
| “Oh goodness, I need to learn”: Specific knowledge gaps | Specifics clinician knowledge gaps in providing transgender health care (e.g., dosing, care options, endocrinology, social issues, language) | “When I started having some patients that were gonna come to me and I was gonna be their primary care provider and they wanted to go on hormone therapy, then it was like, ‘Oh goodness, I need to learn. I actually need to learn the fundamentals, the basis. The endocrinology behind this and good, correct interview format.’” (NP-218)  “[The patient] had all the terms of what to do and words I didn’t even recognize and acronyms of things of feminization, surgery, and things I knew about but like these little words that I had never heard.” (MD-210) | |  |
| “I didn't understand what all it was about”: Knowledge gaps that harm | Reflections on how knowledge gaps contributed to patient harm | “I don’t understand what’s going on but this is what- she’s been to the ER three times, she’s been to Urology once, and now she’s saying she can't urinate and you know and obviously with transgender I mean, it [syphilis] was up her urethra, you know she has a penis but I didn't understand what all it was about, and I still don’t completely... So, I think some was about transgender, I thought some of it might be about reckless sex but I wasn’t sure. I just didn't know what to make, so I was pretty stooped. And then really since the syphilis has been treated, she’s a different person. She feels well and she just looks different.” (PA-227) | |  |
| “That was something new”: Knowledge gaps based on caseload | Characteristics of patient caseload inform knowledge gaps (e.g., age, gender) | “My initial patients were primarily male to female, although it didn't take long before I had my first female to male patient come in. And so then that was something new. Like, okay, well how are we going to do this?” (MD-214) | |  |
| **2.2 She was not comfortable: Establishing a patient-clinician relationship** | | | |  |
| “She was not comfortable”: Reciprocal distrust | Mutual distrust between patients and clinicians | “Some was discomfort with her in telling me about her sexual activity, and I felt really bad about it, but she was not comfortable with that." (PA-227)  “Now everybody knows what the script is supposed to be, so they'll tell you what they think they need to tell you to get hormones, so the history is often not quite valid.” (MD-220) | |  |
| “There's a lot of, you know, different expectations”: Meeting patient expectations | Clinician challenges to meet patient expectations | “There’s a lot of, you know, different expectations. There’s a lot of these transgender patients have seen hormonal therapies or have taken hormonal therapies outside of the medical establishment. There’s a lot of lore about how well they should work and what works best, etc. etc… so there’s an-- sometimes an expectation that, you know, ‘I was on,’ you know, ‘I took Premarin before. Why aren’t you giving it to me now?’” (MD-224) | |  |
| **Theme 3: Power to deny: Prescriptive authority and gatekeeping** | | | |  |
| **3.1 Gatekeeping: The stigma, discomfort and underlying denial of hormone therapy** | | | |  |
| Conditional access to hormone therapy | Conditional access to hormone therapy until engaged in mental health services | Respondent: “ I send all of that to mental health.” Interviewer: “You do?” Respondent: “Yes.” Interviewer: “You said every single patient or—" Respondent: “Yep”. Interviewer: “--just the ones you’re not sure about?” Respondent: “Nope. All of them. Mmm-hmm. Maybe one I started on hormone therapy because the patient had already transitioned on their own and I think it was safer to start her instead of saying, “Hey, you need to go to mental health before you get started.” But the rest of them are people who was like, “I’m thinking about hormone therapy,” and I send them to mental health because based on the psychosocial component of it, I think there are some underlying mental health issues that need to be addressed and I think that’s a good way to getting them in to mental health, and a lot of these are discovered in mental health. You might send them to seek theoretical hormone therapy but in the end there are the things that-- because I think just-- that’s a big decision to make, to change who you are as a person or who you were born with and I think there are a lot of issues that go along with that. Personal issues, family issues. That’s why I like them plugged into mental health so after they start the hormone therapy, they can continue to see mental health to try to figure out how to cope with these issues.” (MD-210) | |  |
|  |  |  |  |  |
|  |  |  |  |  |
|  |  |  |  |  |
|  |  |  |  |  |
|  |  |  |  |  |
|  |  |  |  |  |
|  |  |  |  |  |
|  |  |  |  |  |
|  |  |  |  |  |
|  |  |  |  |  |
|  | Conditional access to hormone therapy based on health status | “One of the potent-- most potent risk factors for clots and heart disease in these patients is smoking, so I won’t prescribe hormonal therapy to people who smoke, and so you have to sort of take their word for it that they’re not smoking. I don’t, you know, measure, you know, cotinine levels in their hair or whatever the, you know, nicotine metabolites, but I think it’s really, you know, there’s a lot of good reasons to stop smoking <laughs>, and this-- and this is one of them... typically what I do is if they’re smoking, I say, ‘You know, I’m happy to prescribe these for you, but I’m not going to do it if you continue to smoke,’ you know, the risks are really that high, and so what I did a few weeks ago with a new transgender patient was to say-- to prescribe the person a nicotine patch, and then they had a follow-up with their primary provider in four weeks or so, and I said, ‘You know, you can get off the-- if you get off the cigarettes when you meet your doctor in four weeks, then we can prescribe the hormones at that point.’” (MD-224) | |  |
|  | Conditional access to hormone therapy based on commitment and desire to fully transition to the opposite sex assigned at birth | “I personally do not prescribe unless someone is committed to living the rest of their life in the gender other than the one they appear to be at birth. So, I don't give hormones if people don’t want to transition or aren't sure if they want to transition. Like if somebody says, ‘Well, maybe if I go on hormones that'll help me know if I want to transition.’ I don't feel comfortable with that.”  Interviewer: “What's the concern?” Respondent: “I believe that there is such a thing as transsexualism that hormones are a treatment for transsexualism that a transsexual is somebody who strongly desires to live their whole life in the other gender. So, I don't prescribe it for other conditions.” Interviewer: “So if someone came to you and said, ‘I'm genderqueer [ph?] I want to take hormones but I don't want to be either gender.’” Respondent: “I wouldn't say you're fucked up, but I would say, ‘I don't prescribe hormones in this situation.’ I'm okay setting that limit. It's a limit it's my limit for my behavior. I guess I feel like I have-- if you don't want to be in either gender why do you need hormones.” (MD-220) | |  |
|  | Conditional access to hormone therapy based on other factors (e.g., requiring a homogenous transgender narrative, already initiated social transition, financial, emotional or housing stability) | “So I have some questions that when I first meet somebody I’ll ask things that I’ve been advised to ask [as a criteria to prescribe hormones] about when did you first know you were trans? Or tell me about your childhood and have you ever taken hormones before? Who are you sexually attracted to?... decided which restroom they’re going to go into. Have they decided what name and feel really comfortable?... And social stability and you know, do they have stable housing? Do they have stable income? How are they gonna pay for their medications I think is important to take into account beforehand...I have to stop myself from prescribing if I don’t feel like they’re-- I don’t want them to take something off the street but at the same time I want them to be emotionally, physically, financially; all those other ways stable before I start because I don’t want to start something that somebody can’t finish or doesn’t want to finish or doesn’t understand.” (NP-218) | |  |
|  | Referring out for hormone therapy | Respondent: “I wasn’t giving hormone treatment.” Interviewer: “Yeah.” Respondent: “I was just a primary care provider.” Interviewer: “Okay.” Interviewer: “ So when they come to you for their primary care would they talk to you about their hormones or where they were getting them or was it not out on the table?” Respondent: “ I think that they somewhat… Basically what I did was tried to refer all those patients to [clinic name].” (PA-212) | |  |
| “I'm not comfortable”: Discomfort providing care | Factors that contribute to comfort in providing care for transgender individuals | “I wasn’t seeing a lot of transgender patients and the ones I were seeing I referred to someone else and said, ‘Look, I’m not comfortable because I don’t know exactly what I’m supposed to do yet,’ and then just referred them to another provider until I became comfortable in terms of actually having a lesson on it... I was like, ‘I’m not comfortable seeing this person. If I don’t know what I’m doing I’m not going to see this person because I won’t be able to treat him appropriately.’” (MD-210) | |  |
| “You’re Doing Something that is Nonstandard”: Collegial and organizational pressure | Organizational pressure against providing gender affirming care | “I specialize in endocrinology, but with transsexual patients I try not even to write the prescription. I will work with their private physician in their local community to do this, and the reason for this is [organization's name] has a bit of a schizophrenic reaction to this whole thing. You know historically that [organization name] was one of the first places to do transsexual surgery in the '60s. Then it became very out of favor, and you couldn't even say the word, so to speak, in this institution...So, anyway, I try not to advertise it so much that I see these patients.” (MD-223) | |  |
|  | Peer pressure against providing gender affirming care | “I got a call from his psychiatrist one day. Basically, ‘What the hell do you think you're doing giving this guy estrogen? He's not transgender. He's just crazy and this is absolutely contraindicated therapy, and I'll make trouble for you if you persist in this.’ Or something like that. And I only ever talked to this guy once. But I thought, ‘Okay, I don't need that.’” (MD-214) | |  |
| “My license is still on the line”: Fear of malpractice | Legal concerns about gender affirming care | Respondent: “It’s not something you will say but a lot of people really are worried about their license and if you feel like you have-- and everything here is about proper training and certification and this and that. So if you’re in the office by yourself and quote unquote you haven’t had some kind of certification or proper training saying you can do transgender care, you may not do it because in the end it really is about your license. As much as people don’t want to admit, it’s the truth with everything we do now.” Interviewer: “That’s very interesting. Because you’re one of the providers that has gotten certified in HIV care. Does that make you feel more secure in providing HIV care?” Respondent: “Not really...I don’t need a certification but I’m comfortable here because I’ve been doing it for so long and I had the guidance, the on-the-job guidance, but if I’m out in suburbia somewhere and I haven’t had any quote training on this then personally I wouldn’t think I would’ve done it either, even if I’m in a city clinic and someone is coming in asking me for hormone therapy and I had no other-- I wouldn’t do it because I think in the end I feel like my license is still on the line for doing something that technically I’m not quote unquote trained to do.” (MD-210) | |  |
| “It's not very reversible”: Fear of hormone permanency | Fear of prescribing hormone therapy due to irreversible effects | “I'm not as worried about prescribing estrogens because they're really pretty darn reversible. And I have had one famous case that reversed. I'm more concerned about testosterone because it's not very reversible. So, I'm really concerned-- I think there are probably an increased cardiac risk with testosterone. It hasn't been proven yet, but I'm very concerned about it. And once people are well transitioned, I try to taper them to the lowest effective dose of testosterone.” (MD-220) | |  |
| “The Complications. You die from it”: Fear of hormone side effects | Fears of prescribing hormone therapy due to potential side effects | Respondent: “I would think that also the physicians don't want to treat. They're afraid, and they want to refer them out somewhere.” Interviewer: “What are they afraid of?” Respondent: “The complications. You die from it. You give somebody high dose estrogens then they're going to have a thrombotic event, and that's where the deaths are. So, they really need to be castrated first, because if you castrate a man, he then has zero testosterone, and then it's easy to add some estrogen. While if the man still has his testes, and is making masculine levels of testosterone, he needs a lot of estrogen to overcome that testosterone. So, a man that is not medically or chemically castrated requires a huge dose of estrogen, and that's associated with gall bladder disease and blood clots. So, I think for that reason a lot of people are scared to treat. I never personally have had a patient that has had a death, but then again, I may not know…Sometimes I'm afraid I'm doing the wrong thing, particularly with young people. I have several young people I follow, and they just make me nervous... Many of the changes that occur are irreversible, and dangerous, and deadly, so sometimes I think that it's not really looked at as seriously as it should be, and I don't know what the answer is” (MD-223) | |  |
| “I'm afraid of being sort of bombarded with this”: Fear of referrals | Fear of becoming known to provide care to transgender patients and consequently overwhelmed with referrals | “I'm not looking for particularly more referrals of this population because I'm afraid of being sort of bombarded with this...So, anyway, I try not to advertise it so much that I see these patients, and one reason is that [healthcare organization name] has this schizophrenic reaction to it [providing care to transgender people]...I don't want this to be my entire focus at all. I see lots of very interesting kinds of patients that I can really help, and I certainly wouldn't want to spend my whole day treating people who are transsexuals.” (MD-223) | |  |
| **3.2 Exceptions to the gatekeeping rule** | | | |  |
| Continuation of hormone therapy | Clinicians continue prescribing hormone therapy for patients already using hormonal therapy | “From my standpoint a mental health profession that doesn't have an interest or experience or knowledge in that area is not going to be able to make a better decision than I am. It's just like passing the buck to someone else than having to take responsibility. I mean there are other things you can do which is try to you know the guidelines, Harry Benjamin Guidelines to live in your identified gender for six months and see how you do. I've done that before too. But it's definitely hard. The thing is though that a lot of the trans patients that I've had come to me already on hormones. And at that point even if do have some discomfort I'm sort of inclined to grandfather them in.” (MD-207) | |  |
|  |  |  |  |  |
|  |  |  |  |  |
|  |  |  |  |  |
|  |  |  |  |  |
| Initiation of hormone therapy | Clinicians’ initiate hormone therapy for patients without conditions | “I guess theoretically there’s supposed to be a protocol that we all follow, but it’s pretty independent. I think I’m probably on the loosey goosey end. I sort of feel like if the person is not crazy and sometimes even if they are crazy but they know what they want, but usually if they’re not crazy, like if they’re not clearly like delusional, <laughs> if they’re in the same space with me and the same reality and they’re clear about what they want and we’ve had at least another visit or two to talk about and think about all the implications of the changes, what they’re prepared for, then I guess it would be more like an informed consent model where I think the person knows what they’re doing, they feel like they know what they’re doing, we go over the risks and benefits, and then I’ll prescribe. Some people aren’t clear and they ask for a therapy and so then I connect them with therapy. Some people come in already on hormones and then I just continue them. <laughs>. Some people come in with a letter already from a therapist. But I have to say I can’t think of a time where I said somebody absolutely had to have a mental health visit before I prescribed and the time I think that that would be true is somebody I was really worried about having significant either ambiguity or some non-reality about what to expect or somebody with serious mental illness and I was worried about how some of the emotional changes associated with hormone therapy might affect their underlying mental health and would want to make sure that they were connected before we did that.” (PA-200) | |  |
|  |  |  |  |  |
| **3.3 Power to prescribe or deny** | | | |  |
| Recognition of power of prescribing | Reflections on the inconsistencies and provider judgment in facilitating or creating barriers to accessing hormone therapy | “There’s the World Professional Association, the HB, the Harry Benjamin. The Harry Benjamin standards are carrying the 2001 standards. But they can be very differently interpreted. I mean some people feel like the standards say you have to have a real-life test, meaning live for six months to a year, I think three months is the shortest, in your chosen gender identity, and that was chosen with quotes, before hormones or before surgery, and for most people, that’s not possible even if they wanted to do it. And some people feel like that’s not necessarily a requirement before hormones because it would be difficult to do it without hormones. Some people feel like the mental health requirement is a requirement and some people feel like it’s a suggestion, so I think there’s all different ways of interpreting it. So while there are standards, I think they’re interpretable standards and I think that, you know, in the 10 years since they were written, I think they were very progressive at the time, but that the community has moved forward and health- that even the notion of sort of informed consent in healthcare has moved forward since then. So I’ll be very interested to see what the new guidelines say... I think because the medical officer usually trains people who haven’t done trans health before, people tend to follow the model that she follows, which is closer to the standards of care [reference to the Harry Benjamin standards], not entirely, but closer to the standards of care. But I mean for me, I felt like I already had a way of doing it before I got there and unless somebody tells me I can’t do it that way, I’ll probably just keep doing it. <laughs>.” (PA-200) | |  |
|  |  |  |  |  |
|  |  |  |  |  |
|  |  |  |  |  |
|  |  |  |  |  |
|  | Reflections on clinicians’ power to prescribing hormone therapy and influence colleagues to provide transgender health care | “And sort of like, who am I treating here if I insist on saying, ‘Why do you want this? And unless you meet criteria, I'm not going to do it.’ And so, I've had to ask myself sometimes over the years, well, is that my sort of bias? And because I'm a doctor and I have the privilege of prescribing things, and that power, which I can either exercise or not exercise, who am I doing it for? Is it my intellectual rigidity that says, ‘Well, by my definition, you're not really transgender and therefore you don't get the hormones.’ But this one, ‘Yeah, you fit and so it's okay for me to give them to you.’ So I don't know. There are still areas which are sort of a little unclear.” (MD-214) | |  |
|  |  |  |  |  |
|  |  |  |  |  |
|  |  |  |  |  |
| **Theme 4: Stigma: This is really strange, and I can’t really understand it** | | | |  |
| **4.1 Negative stereotypes: They’re really homosexuals that are afraid to admit it** | | | |  |
| “Risky sexual behaviors" | Stigmatizing beliefs about the sexual behaviors of transgender people | Respondent: “But there is kind of a larger percentage of trans patients that [engage in risky sexual behaviors that] is job related. They’re doing drag shows and maybe stripping and stuff like that. That’s unfortunate too though.” Interviewer: “Yeah. What do you think leads people to do that?” Respondent: “I think it’s the attention they’re getting.” Interviewer: “Oh, the attention.” Respondent: “And I think it’s being accepted in that group of people who are observing them.” Interviewer: “Hmm.” Respondent: “Yeah. And they’re looking for it in the wrong places obviously”. (PA-212) | |  |
| “They're really homosexuals that are afraid to admit it”: Homophobia | Stigmatizing beliefs about the sexual orientation of transgender people | “A lot of people here in psych think that they're really homosexuals that are afraid to admit it, and so I don't know how much of that it is.” (MD-223) | |  |
| “These can be difficult patients”: Difficult patients | Stigmatizing beliefs about the mental health of transgender people | “These can be difficult patients, particularly trans women often have had pretty rough experiences and are pretty rough people and not necessarily compliant with visits or medications, follow up. And I swear there's a higher incidence of personality disorders among trans women so it can be a difficult group. I have no way of knowing. It could be anything from environmental stresses to something that's linked to whatever gene causes transgenderism.” (MD-220) | |  |
| **4.2 Physical appearance: It’s this obsession** | | | | |
| Physical appearance preoccupation | Stigmatizing beliefs about obsession on physical appearance | “And to me I have my point at which I say "Okay you look great. You look like a woman so let's get on with your life already." You know so it's not I don't have a problem with a trans woman wanting to look like a woman. But some of my experience with patients is it's this obsession and it's- like it's never going to be okay. And I understand where that comes from but I think it's more of a psychological problem. We all have to draw the line with a lack of perfection.” (MD-207) | |  |
|  |  |  |  |  |
|  |  |  |  |  |
| Passing prejudice | Stigmatizing beliefs about transgender people that are not perceived as a cisgender person | “I think that sometimes it's a-- it's very difficult to transition, particularly the male to females are very hard. They never quite get the mannerisms of women, and it always looks artificial. You can spot them a mile away. The females to males are scary how good they do, and how they completely pass… The males to females they just are always so awkward. They have a lot of the masculine features, the square jaw, the brows, that even when they're taking hormones they don't fully pass. The really good patients I have, good from the point of view of how they look and how they behave, they've done more than just genital surgery. They've softened the jaws. They change things with the face but, again, these things cost money.” (MD-223) | |  |
|  |  |  |  |  |
|  |  |  |  |  |
|  |  |  |  |  |
|  |  |  |  |  |
| **4.3 This is really strange, and I can’t really understand it** | | | | |
| Discomfort | Anticipation that other clinicians will be uncomfortable with transgender people | “So, it all worked out and the person [provider] who saw her [the patient] at the other was completely fine with it [the patient being transgender].” (PA-227) | |  |
|  |  |  |  |  |
| Disbelief | Disbelief or lack of understanding of transgender people | “I think if I asked him, and I do, I think he's happier, probably, and the data show that people that do transition are happier, but I just looked at him and felt to myself, ‘Are you really happier? Everybody's getting married and having children now and you're still in this little limbo state a little bit.’ So, I don't know. I don't know, sometimes I see people and say, 'I just don't understand it.’...Many of the changes that occur are irreversible, and dangerous, and deadly, so sometimes I think that it's not really looked at as seriously as it should be, and I don't know what the answer is. Sometimes I feel like saying to somebody, ‘Can't you grow out of this a little bit,’ but yet I see people suffer from it as well... part of me wants to sort of say like, ‘Just you can't dress as a woman,’ or ‘Can't you just be a tomboy and not have to get involved with hormones and stuff?’” (MD-223) | |  |
| Discrimination | Observed discrimination against transgender patients in healthcare | Respondent: “So, I had a lot of transgender patients that would come through there and I would personally try to take them as patients so that they wouldn’t be discriminated or laughed or ridiculed.” Interviewer: “That’s actually really interesting to me. So, what would happen typically in the E.R. when a transgender patient would come in?” Respondent: “A lot of laughing and snickering and people would walk in just to see them like it was a sideshow type of thing. The doctors would flip a coin over, like, who had to take them, literally.” Interviewer: “So, they didn’t want to see them?” Respondent: “No, they didn’t want to see them. And the same with the HIV patients. And was just very, you know, made the patient feel very uncomfortable. And I didn’t feel that the treatment that they received was the same as a person coming in with the same problem that wasn’t transgender. It was just unfair about allowing visitors to come in and things like that.” Interviewer: “A lot of visitors would come out?” Respondent: “Yeah. You know, it could be their partner but because they weren’t married or their immediate family member, stuff <laughs> like that." (PA-212) | |  |
| **4.4 Go all the way** | | | |  |
| Goal of invisibility | Stigmatizing beliefs about gender non-conformity | “I try to get patients to be realistic. If you want people to accept you in the chosen gender you have to be invisible realistically. And most people can be invisible and there's a good number who are invisible that haven't changed their names, I don’t get that. In other words, I don't put-- I'm more of a realist. If you want to transition, go all the way and transition. Probably, except for issues involving the genitals because most people haven't had surgery, otherwise there's no reason if you change your name for anybody to know…I really think the best thing for a trans person is to transition as fully as possible as soon as possible. The more transitioned they are the more completely transitioned the better things are going to get. If that doesn't work then they're not exactly transsexual they're something else, which has its own set of problems. Like identifying as genderqueer is-- my, this is politically incorrect. It's a socially defiant stance because everybody in the world wants to identify you as male or female, everybody except the six other genderqueer people and a group of sympathizers. So, it's like setting yourself up in opposition and that's why people usually get over it by the time they get out of their 20s. And if not, they may have some other pathology personality disorder or something. I know these are strong statements, but I view transsexuals as a normal variant, it's a condition, the treatment is transition. And if transition isn't the treatment, then that's not the diagnosis. It's not transgenderism if transition doesn't work for it. And then you have to question how is-- other than to try to cut down prejudice barriers, what is the role of the doctor. I don't think there's a prescribing role there.” (MD-220) | |  |
|  |  |  |  |  |
|  |  |  |  |  |
|  |  |  |  |  |
|  |  |  |  |  |
|  |  |  |  |  |
|  |  |  |  |  |
| **Theme 5: Reflections: Strategies for success, rewards, and personal motivations** | | | |  |
| **5.1 Becoming proficient: Strategies for success** | | | |  |
| Patient-centered care | Creating patient-clinician partnerships through getting to know the patient | “[Discussing the first patient visit] …so if there’s time I think it’s a good opportunity to get to learn a little bit about what these-- what these people are facing, you know, and what they’re, you know, they’re-- the struggle that they have-- they live with and acceptance and people’s perception of them.” (MD-224) | |  |
|  | Creating patient-clinician partnerships through prioritizing patients’ goals | “Well, the first thing I’m going to do is just establish what their goals are, what they would like to accomplish. And just try to really let them feel comfortable in opening up and feeling that no one’s going to be judgmental. And then just to try to find out how they’ve been perceived in the community and what’s their relationship like with their families, what are their career goals, things like that.... And what do you know so far? What are your expectations? And then after we’ve gone through that then I tell them basically what they can expect.” (PA-212)  "Usually the visits are patient-driven. You know, the first thing I ask is, ‘What’s the most important thing for you to get done today?’ so that we make sure we do that. <laughs>. We leap in." (PA-200)  “We go straight where their needs are. In adolescent health we have to really understand what is the agenda of the patient. So my agenda has to change according to what the patient really needs that day...Because if they <inaudible> estrogen, and that's all they want to talk about, then I try to use the opportunity to talk about the other step. ‘Are you taking your other medicine, and how is that going? Is it giving you anything, or is it so hard?’ Then like that I accommodate into their style and then teaching them the preventive tools.” (MD-239) | |  |
|  | Creating partnerships through communicating clear, honest, and realistic expectations | “I really like how on one of our consent forms; I think it’s the consent form. It talks about how this is like almost a five-year process and I don’t know where I’m coming up with the number five but with the idea of puberty didn’t happen overnight and this is a hormonal change and this isn’t gonna happen overnight. You might see changes immediately, but other things are gonna be over time. And so working through that transition, I mean, the stress of moving to a new city can have people on a rollercoaster. Well, how about the stress of changing your name and changing your gender and changing how you’re viewed on the street or how you view yourself. You know, that can send people on a huge emotional rollercoaster.” (NP-218)  “I don't have any standardized basis for how to do this. But I will be happy to work with you and we will sort of do things together step by step and see what the results are, and make sure there are no problems or toxicities and what have you.” (MD-214) | |  |
| Time/practice | Reflections on increased proficiency and competency with increased time and practice | “I have gained a lot of comfort with the sex hormones, their administration and their safety over the years, just based on my cumulative practice.” (MD-214)  “Once you do it, you kind of just- you just get better at it.” (NP-213) | |  |
| Clinical environment | Reflections on how ones’ organizational environment and culture facilitates care delivery to transgender people (e.g., diverse and culturally competent staff, no restrictions on medications or limits on visit duration, and systems/templates that support patient care) | Respondent: “If you would be in kind of a suburban or even urban clinic where you really depend on the billing that you’re going to make and you need that person to show up and you know, every 15 minutes, that’s what you do because you need to do that. But here, it’s a little different. That’s why we bring that funding to be able to give us that flexibility to say, ‘I’m going to talk to you.’” (MD-239)  Respondent: “Well, in our clinic, everybody is really very, very sensitive and competent, culturally competent to deal with trans-genders and sexual minority youth. This is how we hire.”  Interviewer: “Oh, so it is something you screen for or a training you provide?” Respondent: “Absolutely.” Interviewer: “So, you screen for it, but—" Respondent: “Absolutely, absolutely. And not only that, the youth screen for it. So, the Community Advisory Board is part of the interview committee.” Interviewer: “Ah, that’s exciting. And the Community Advisory Board is made up of youth?”  Respondent: “Yes. Youth.” (MD-239) | |  |
| “Facing their own fears and getting comfortable treating different types of people”: Confronting fears, and biases | Reflections on grappling with the benefits and risks of hormone therapy | “I think a lot of people are sort of accepting of the fact that this is a real issue for people, and it’s a real quality of life issue, and it’s an important one to get right and to be able to balance some of the risks and benefits is important… The goals of therapy are quite different, right? So in someone who has diabetes, for example, you are trying to improve their metabolic profile to prevent a bad outcome of cardiovascular disease, retinopathy, kidney disease or nerve damage, and so you always sort of have that in the back of your mind that sort of that’s what you’re doing, so for-- you don’t really have that with transgender therapy, and you are thinking, ‘Okay, well I’m improving this person’s quality of life by helping them feel that-- the way that they want to feel.’ A lot of that has-- is-- has to do, you know, you’re getting feedback instead of looking at lab tests to be sure their hemoglobin A1C is better and their lipids are well-controlled, that they have, you know, your microalbumin. You’re asking them how do they feel, and so-- and then you’re balancing it against the risk side of things, and so you know that if you were to double their estradiol, they might feel a little bit better, but you also know that it may open them up to more risks, and so there’s not a clear sense, you know, because their risks aren’t very well-known, and people don’t-- you know, you can talk about numbers, but, you know, it might not strike home to them that the risk of having a DVT is one in 1,000, so what does that really mean? Some of these tradeoff questions would probably be better. ‘Would you be willing to have a clot or pulmonary embolus?” “How much would you be willing to sacrifice to feel the-- to get the hormones?’ So, the goals of therapy are sort of fundamentally different than some of the other things that we do.” (MD-224) | |  |
|  | Reflections on evolving comfort with providing care to transgender people | “It took really kind of facing their own fears and getting comfortable treating different kinds of people that they had not seen in their training. And I saw people really move very far in their beliefs and understanding.” (PA-227)  “Some initial discomfort with the whole concept of transgenderism, which I think I made a good evolution to feeling comfortable... I think more than other alternative sexuality issues with patients, that alternative gender is another level of discomfort for everybody. And I had some of that, I think. It was like, this is really strange. And I really can't understand it. But we're taught to believe patients. And it was quite clear from early on, these weren't crackpots. They were very sincere and really needed some help and really wanted to transition to a different morphology and life and everything. So whatever initial discomfort I may have had sort of faded away.” (MD-214) | |  |
|  | Reflections on personal biases and the need to face personal biases | Interviewer: “You train new doctors. What is the most important message you want them to learn?” Respondent: “Tolerance.” Interviewer: “Yes?” Respondent: “Yes. I think it’s not really tolerance, but it’s really being competent in the care of a patient and seeing the patient as an individual not as how they look like or you know, who are you perceiving they are. It’s really looking at the patient. That’s, I think, and that brings all the, you know, the empathy and the taking away the biases that we bring. But I really say to them, take a look at that human being because that human being can be so charming, even in their acting out because they’re bipolar and at this time they are so hyper. But that person can bring a lot, and they can bring a lot into their families. So, you know, just look at the person first. And second, look at your own biases, what you bring into the table. But really, do you know focusing on the patient as a human being, as a person, I think it makes a world of difference, you know, for a physician, because then they don’t think it’s them.” (MD-239)  “I think specifically as a lesbian there was a sense that, you know, you get this body, it’s a woman’s body and then what it means to be a woman is how you live. And you can live in a woman’s body, in a female body and be a very, very masculine woman and that was part of my politic. So it was a challenge to me to think that you could be born in a female body and instead of fighting, or maybe in addition to fighting to have the world be a more accepting place of all the ways it is to be a female body including a very masculine woman, that there were people who were choosing to change their body instead of the world <laughs> I guess was how I thought about it. And, you know, that was hard for me to understand... I think that feeling is fine for me. Like that’s how I approach it. Like I’m in this body, I’m fine with it, I want the world to be more accepting of how I act, dress, look in this body, whatever that is...my own stuff around this societal push to be a beautiful woman, like if you’re a woman you have to be a beautiful woman and beauty means this and then to see people trying so hard to achieve that, ‘cause I’ve spent a lot of my own emotional energy trying to reject that. <laughs>.” (PA-200) | |  |
|  | Reflections on fear of failure, particularly for clinicians early in their careers | “I think young people who are in training, they are so fearful of failure, and I try to take that away from them and say, you’re not failing. You have done what you can and you’re going to learn even better how you do it, but you’re going to learn even better how you do it, but it’s not your failure.” (MD-239) | |  |
|  |  |  |  |  |
| Mentorship from within | Reflections on importance of a mentor at work | “I usually run it past my medical director and I’ll have heard read through my note and say how did I do? Did I ask the right questions and what do you think about this presentation?” (NP-218)  “We communicated in flags, but I'll call her every now and then but flags and she responds very quickly and she's always happy to answer... I think it would be difficult not having her there.” (NP-213) | |  |
| **5.2 Why I provide care** | | | |  |
| “You notice how happy they are”: Rewards and benefits | Reflections on the benefits and rewards of providing care to transgender people | “At least you know you’re providing care in a place where a patient will come and be comfortable talking to you about whatever, whatever issues they have without you judging them...secondly you see the transition of how the person changes. Like how they’re happier or they’re into that process of changing. I don’t know, like you notice how happy they are.” (MD-210)  Respondent: “I feel that I am instrumental in helping them achieve in most cases lifetime goals for their selves. And after being there almost two years now, being able to see the difference from the time they first started until now and after almost two years and being able to also see how it’s changed their lives, the quality of life is different.” Interviewer: “How so?” Respondent: “They’re more accepting of their selves, their self-esteem is greater. They’re more out in the community. They’re more productive at working now and they’re not struggling anymore, ‘What should I do?’ Decision’s been made, I’ve been treating them. And they really like the way they’re looking. And that to me is exciting.” (PA-212)  “I feel that this is something where she’s [the patient] given me a lot too, you know this is kind of one of those things where you feel like it’s mutual.” (PA-227) | |  |
|  |  |  | |  |
| “All I see in front of me is someone who is sick, and I just need to treat them”: Doing my job | Reflections on humanizing transgender people as people | “I know we have transgender patients, and I never knew what that would be, but I remember my very first patient ever was a transgender female and I think at that moment I just realized that a person is a person. That’s what it is. You’re not there to look at the person, you’re just there to treat them for who they are as a patient. So, I think that was my very first experience and then it teaches you. We all say we’re humble and all the other good stuff they tell you you’re supposed to do and say. You know, treat everybody the same, all that good stuff. But until you actually have that moment where it’s like ‘Wow, I am sitting here with someone that doesn’t look like me and have many choices based on who knows what in regards of whatever views I may or may not have had or never had this experience, all I see in front of me is someone who is sick, and I just need to treat them.’ So that was my first experience, first patient ever.” (MD-210) | |  |
| “Tikkun olam” | Reflections on a desire and commitment care for underserved communities | “I just had this feeling that I didn't want to spend my time taking care of people that seemed so over privileged...I was interested in just serving a population that needed to be served. Kind of the Jewish Tikkun olam, try and help the world, stuff.” (MD-215)  “I'm chronically interested in underserved people, same way I got interested in HIV. So just whatever gaps appear.” (MD-220) | |  |
