# Supplementary material for: Interdisciplinary clinicians’ attitudes, challenges, and success strategies in providing care to transgender people: a qualitative descriptive study
Source: BMC Health Serv Res. 2022 Sep 8;22:1134. doi: 10.1186/s12913-022-08517-x (PMC9454229; doi:10.1186/s12913-022-08517-x)
Supplement: Supplementary file 4 — Additional file 4. [file 12913_2022_8517_MOESM4_ESM.docx]

| **Saturation table of themes and subthemes** | | | | | | | | | | | | | |
| --- | --- | --- | --- | --- | --- | --- | --- | --- | --- | --- | --- | --- | --- |
| **Subthemes** | **Medical Doctors** | | | | | | | | **Nurse Practitioners** | | **Physician Assistants** | | |
|  | **207** | **210** | **214** | **215** | **220** | **223** | **224** | **239** | **213** | **218** | **200** | **212** | **227** |
| **Theme 1: Knowledge acquisition: Formal & informal pathways to competency** | | | | | | | | | | | | | |
| Formal pathways to competency |  | x | x | x | x |  |  | x | x | x | x |  | x |
| Informal pathways to competency | x | x | x | x | x | x | x |  | x | x | x | x | x |
| **Theme 2: Perceived challenges and barriers: *I didn’t know what I was doing*** | | | | | | | | | | | | | |
| *People don’t know how to treat:* Knowledge gaps in providing care | x | x | x | x |  | x | x |  | x | x | x | x | x |
| *She was not comfortable:* Establishing a patient-clinician relationship |  | x |  |  | x |  | x | x | x | x |  |  | x |
| **Theme 3: Power to deny: Prescriptive authority and gatekeeping** | | | | | | | | | | | | | |
| Gatekeeping: The stigma, discomfort, and fear underlying denial of hormone therapy | x | x | x | x | x | x | x | x | x | x |  | x |  |
| Exceptions to the gatekeeping rule | x |  | x |  | x |  | x | x |  |  | x |  | x |
| Power to prescribe or deny | x |  | x | x | x |  |  |  | x | x | x |  |  |
| **Theme 4: Stigma: *This is really strange, and I can’t really understand it*** | | | | | | | | | | | | | |
| Negative stereotypes: *They’re really homosexuals that are afraid to admit it* |  | x | x |  | x | x |  |  | x | x |  | x | x |
| Physical appearance:  *It’s this obsession* | x |  | x |  | x | x |  |  |  |  |  |  | x |
| *This is really strange, and I can’t really understand it* |  |  | x |  |  | x |  | x |  | x |  | x | x |
| *Go all the way* |  |  |  |  | x | x |  |  |  | x |  | x |  |
| **Theme 5: Reflections: Strategies for success, rewards, and personal motivations** | | | | | | | | | | | | | |
| Becoming proficient:  Strategies for success |  | x | x |  | x |  | x | x | x | x | x | x | x |
| Why I provide care | x | x | x | x | x |  |  | x |  | x |  | x | x |

*Note*: The red ‘x’s indicate the first time a subtheme was identified among the interviews to provide evidence of data saturation. The black ‘x’s indicate each instance a subtheme was identified among all interviews, to provide a summary of thematic prevalence or the breadth of each subtheme across the entire study.
